# Epicardial Adipose Tissue, Adiponectin and Leptin: A Potential Source of Cardiovascular Risk in Chronic Kidney Disease

**DOI:** 10.3390/ijms21030978

**Published:** 2020-02-01

**Authors:** Luis D’Marco, Maria Jesús Puchades, Jose Luis Gorriz, Maria Romero-Parra, Marcos Lima-Martínez, Carlos Soto, Valmore Bermúdez, Paolo Raggi

**Affiliations:** 1 Nephrology Department, Hospital Clínico Universitario, INCLIVA, University of Valencia, 46010 Valencia, Spain; luisgerardodg@hotmail.com (L.D.); chuspuchades@gmail.com (M.J.P.); jlgorriz@gmail.com (J.L.G.); Romero.parra.maria@gmail.com (M.R.-P.); 2 Physiologic Sciences Department, School of Health Sciences, Universidad de Oriente, Bolívar 5110, Venezuela; marcoslimamedical@hotmail.com; 3 Nephrology Department, Consorci Sanitari del Alt Penedes-Garraf, 08800 Barcelona, Spain; carlossotomedico@hotmail.com; 4 Facultad de Ciencias de la Salud, Universidad Simón Bolívar, Barranquilla 080005, Colombia; valmore@gmail.com; 5 Mazankowski Alberta Heart Institute, School of Medicine, University of Alberta, Edmonton, AB T6G 2B7, Canada

**Keywords:** Adiponectin, leptin, epicardial adipose tissue, cardiovascular disease

## Abstract

The importance of cardiometabolic factors in the inception and progression of atherosclerotic cardiovascular disease is increasingly being recognized. Beyond diabetes mellitus and metabolic syndrome, other factors may be responsible in patients with chronic kidney disease (CKD) for the high prevalence of cardiovascular disease, which is estimated to be 5- to 20-fold higher than in the general population. Although undefined uremic toxins are often blamed for part of the increased risk, visceral adipose tissue, and in particular epicardial adipose tissue (EAT), have been the focus of intense research in the past two decades. In fact, several lines of evidence suggest their involvement in atherosclerosis development and its complications. EAT may promote atherosclerosis through paracrine and endocrine pathways exerted via the secretion of adipocytokines such as adiponectin and leptin. In this article we review the current knowledge of the impact of EAT on cardiovascular outcomes in the general population and in patients with CKD. Special reference will be made to adiponectin and leptin as possible mediators of the increased cardiovascular risk linked with EAT.

## 1. Introduction

There is a growing interest in the role that visceral adipose tissue plays in the pathophysiology of cardiovascular disease. Epicardial adipose tissue (EAT) in particular has been the target of intensive investigation because of its proximity to the coronary arteries and myocardium and its potential influence on the development of cardiovascular disease (CVD) in the general population as well as high-risk populations [1]. EAT is a rich source of free fatty acids and is capable of secreting proinflammatory and proatherogenic cytokines as well as antiatherogenic adipocytokines. Several investigators put forth the hypothesis that EAT may be capable of promoting atherosclerosis of the epicardial coronary arteries through local paracrine effects [2].

EAT can be quantified by transthoracic echocardiography, cardiac computer tomography (CCT) and cardiac magnetic resonance, although CCT is more accurate and reproducible than other methods [3,4,5]. In early publications, EAT volume was reported to be associated with the presence and severity of coronary artery calcium (CAC), a marker of atherosclerosis. In subsequent studies, a close association with indices of plaque instability, as detected by CCT angiography, was also reported [6,7]. Recent publications have shown a correlation of EAT with coronary artery disease (CAD) and adverse cardiovascular events in the general population [8,9] and patients with chronic kidney disease (CKD) [10,11] independently of other risk factors.

This review explores the potential role of two adipocytokines produced by EAT, adiponectin and leptin, as modulators of CVD in patients affected by chronic kidney disease (CKD).

## 2. Cardiovascular Risk in Chronic Kidney Disease

Numerous studies have shown that a reduction in estimated glomerular filtration rate (GFR) is associated with increased frequency and severity of CVD [12]. Data from a meta-analysis that included 1.4 million patients revealed that, after adjusting for traditional cardiovascular risk factors and albuminuria, CV disease morbidity and mortality increased linearly with a drop in GFR <60 mL/min/1.73 m^2^ [13]. In a Canadian cohort study, after adjusting for age and sex, CVD accounted for 27.5% of deaths in individuals with normal renal function versus 58% in those with CKD [14]. In young adults, as CKD progresses from stage 2 to 5, life expectancy is shortened by 1.3, 7.0, 12.5 and 16.7 y as a result of increasing rates of CVD. Additionally, kidney transplant does not completely correct this loss of life years [14].

The two most frequent clinical features of CVD in patients with CKD are CAD and left ventricular hypertrophy and dysfunction [15]. A combination of traditional risk factors and other factors more closely related to progressive loss of renal function contribute to the high incidence of cardiovascular complications observed in these patients [16,17]. In this context, the identification of patients at risk of cardiovascular events represents a real challenge. Therefore, there is a growing interest in the development of diagnostic approaches that may help risk-stratify patients with CKD, in the hope to improve outcomes by focusing on more aggressive treatments in those at higher risk.

## 3. Epicardial Adipose Tissue

**Figure 1:**
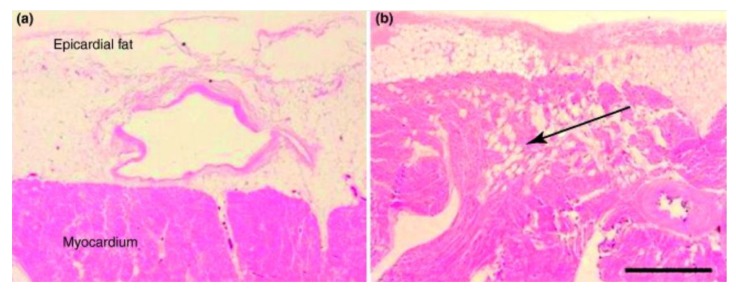
Panel (**a**) the epicardial fat layered directly on the surface of the left ventricular myocardium without a fascia. A cross-section of a small coronary artery is seen embedded in the epicardial fat. Panel (**b**) the epicardial fat is layered on the surface of the free wall of the right ventricle, but numerous adipocytes are also infiltrated among the myocardial fibers (black arrow). Bar scale: 1 mm. Reproduced with permission from [19].

EAT is composed of visceral fat located below the visceral pericardium and in direct contact with the coronary arteries (Figure 1) [18]. In the embryonic stage, EAT develops from brown adipose tissue [19]. Pericardial fat instead lies on the outer surface of the parietal pericardium. Although they are in each other’s vicinity, these two fat deposits are very different. EAT is vascularized by branches of the coronary arteries, while pericardial fat is vascularized by noncoronary arteries. EAT originates in the splanchnopleural mesoderm, while pericardial adipose tissue derives from the primitive thoracic mesenchyme [20]. Of importance, there is no layer or fascia that separates EAT from the underlying myocardium and coronary arteries; this potentially allows a direct diffusion of the adipose tissue contents to the coronary arteries and myocardium. Small amounts of epicardial fat can also be found amidst myocardial fibers generally along the intramyocardial branches of the coronary arteries [21]. The functional role of EAT is complex and incompletely understood, although it probably has multiple functions such as mechanical, metabolic, thermogenic and endocrine/paracrine functions [19].

EAT typically accompanies the main branches of the coronary arteries in the atrioventricular or interventricular groves. It is compressible and elastic and provides mechanical protection of the coronary arteries against excessive deformation during the cardiac cycle [21]. EAT is not simply a passive storage for lipids, but it is actively involved in lipid homeostasis and energy production. In fact, it has a higher rate of free fatty acid (FFA) release and absorption compared to subcutaneous fat deposits and other visceral fat deposits [18]. Since myocardial metabolism is heavily dependent on FFA oxidation, EAT helps to support myocardial energy needs, especially during periods of high demand [22]. Brown adipose tissue contains mitochondria with large amounts of uncoupling protein-1 (UCP1) that is needed to generate heat in response to exposure to cold. Sacks et al. found that expression of UCP1 and its related genes are higher in EAT than in other fat deposits in the body, such as the abdomen, thighs and subcutaneous tissue [23]. These data suggest that one of the functions of EAT may be to produce heat to protect the myocardium and coronary arteries from hypothermia [24].

**Figure 2:**
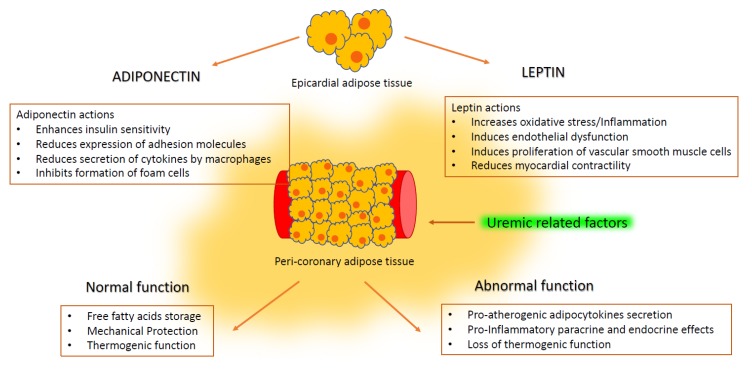
Proposed mechanisms through which adiponectin and leptin may cause cardiovascular damage.

Human investigations have shown that EAT can secrete multiple cytokines involved in the regulation of endothelial function, coagulation and inflammation locally and systemically **(**Table 1) [25]. Several bioactive molecules secreted by EAT can either protect or negatively affect the health of the myocardium and coronary arteries (Figure 2). Under normal physiological conditions, EAT secretes cytokines with anti-inflammatory and antiatherosclerotic functions such as adiponectin [26]. A product of adipocytes, adiponectin has been described as having antidiabetic, antiatherogenic, antioxidant and anti-inflammatory properties [27]. Adiponectin increases the oxidation of fatty acids via a protein kinase pathway and reduces lipid deposition in the myocardium. Additionally, adiponectin inhibits the production of mediators of inflammation and maintains an anti-inflammatory microenvironment in the cardiovascular system [28]. In pathological conditions, the production of adiponectin by EAT decreases while the secretion of proinflammatory or proatherogenic factors (such as leptin) increases [29]. Among the proatherogenic effects attributed to leptin are induction of hypertension, oxidative stress, endothelial dysfunction, inflammation and proliferation of vascular smooth muscle cells [30,31].

## 4. Epicardial Adipose Tissue in Renal Disease

Although EAT has emerged as a risk marker for CAD and has been extensively investigated in the general population, studies in patients with CKD are still limited. In early publications in the general population, EAT volume was reported to be associated with the presence and severity of CAC, a marker of atherosclerosis [32]. In subsequent studies, a close association with indices of plaque instability, as detected by CCT angiography, was also reported [6,7]. Similar findings have been reported more recently in patients with CKD [33,34]. In a study performed in patients with end-stage kidney disease (ESKD) awaiting transplant, the investigators showed an independent association of CAC and EAT with inducible myocardial perfusion defects on nuclear cardiac stress testing [35]. While both EAT and CAC were predictors of an abnormal perfusion scan, none of the traditional cardiovascular risk factors were.

Additional studies showed a larger accumulation of EAT in patients receiving peritoneal dialysis or hemodialysis than that in controls, and an increased EAT volume in the presence of the malnutrition, inflammation, and arteriosclerosis (MIA) syndrome [10]. Two studies to date reported on the association of EAT with adverse outcomes in CKD. In the study by Cordeiro et al. [11], after a median follow-up of 32 months, EAT was predictive of fatal and nonfatal cardiovascular events in 277 patients with CKD 3-5. Although the predictive power of EAT was independent of other measures of adiposity, it added a negligible predictive power to traditional cardiovascular risk factors. In a subanalysis of the RIND trial in 109 de novo hemodialysis patients, EAT was an independent predictor of all-cause mortality after a median follow-up of 4 y [36]. Thus, it appears that EAT is a marker of risk in patients with CKD as well as the general population. These findings may not come as a complete surprise since patients with CKD are known to be in a state of chronic smoldering inflammation. Therefore, an inflamed visceral adipose tissue in direct contact with the heart and coronary arteries may be one of the overlooked links between CKD and the high cardiovascular morbidity and mortality observed in these patients.

## 5. Adipocytokines and Vascular Disease

The interaction of adiponectin and leptin with the cardiovascular system is complex and at times seemingly conflictual. Several studies showed that adiponectin expression is significantly lower in patients with CAD and metabolic syndrome [37], and low adiponectin levels are currently considered a risk factor for CAD [38]. Adiponectin rapidly accumulates in the subendothelial space of an injured human artery. In this location, adiponectin may reduce the expression of adhesion molecules by endothelial cells in response to inflammatory stimuli, suppress cytokines production by macrophages as well as decrease lipid accumulation in monocyte-derived macrophages [39,40].

Leptin is expressed in the periconorary adipose tissue in high concentration, and some data suggest that regional secretion of leptin and other adipocytokines may reduce myocardial contractility. This could constitute another possible link between epicardial adipocytokines and cardiac dysfunction [41]. The expression and secretion of leptin by adipocytes and cardiomyocytes is induced by IL-6 and inhibited by TNF-a. At the same time, adiponectin limits the ability of TNF-a to induce expression of adhesion molecules. Hence, the adiponectin/leptin ratio has been suggested as a marker of dysfunctional adipose tissue and related to cardiometabolic risk factors [42].

## 6. Adiponectin in Renal Disease

Evidence is accumulating that local and systemic inflammation is supported by the adipose tissue via paracrine and endocrine mechanisms [43]. Adipocytokines secreted by the adipose tissue can have both pro- and antiatherogenic activities, and they can mediate the development of atherosclerosis by affecting endothelial function and promoting plaque destabilization [38]. Adiponectin and leptin are the product of mature adipocytes [40,44]. Adiponectin enhances insulin sensitivity [45,46], and likely exerts antiatherosclerotic activities by suppressing the release of proinflammatory cytokines, such as TNF-α and IL-6, and stimulating the release of anti-inflammatory cytokines such as IL-10 [40]. Low adiponectin levels have been observed in patients with obesity, metabolic syndrome, diabetes mellitus, hypertension and established CAD [47]. In contrast, plasma levels of adiponectin in patients with CKD are increased up to three-fold compared to physiological levels, most likely because of reduced clearance and/or catabolism [48].

Observational studies linked low adiponectin levels with CVD both in the general population [49] and in CKD [50]. Zoccali et al. reported that plasma adiponectin levels were 2.5 times higher (*p* < 0.0001) among patients receiving hemodialysis (15.0 ± 7.7 µg/mL) than healthy controls (6.3 ± 2.0 µg/mL), but the adiponectin levels were significantly lower in the hemodialysis patients who developed cardiovascular complications than those who remained free of events [51]. The increased risk of cardiovascular outcomes in CKD patients with lower adiponectin concentrations relative to other CKD patients was unchanged after adjusting for multiple traditional and CKD-specific risk factors. Each 1 µ/mL increase in adiponectin concentration was associated with a 3% reduction in risk of cardiovascular events. Similarly, Becker and co-workers evaluated 227 nondiabetic patients with mild to moderate CKD and 76 healthy subjects matched for age, sex and body mass index [52]. After a mean follow-up of 54 months, they concluded that low plasma adiponectin levels were predictive of cardiovascular events.

In contrast, a subanalysis of the Modification of Diet in Renal Disease (MDRD) database performed in 820 patients with CKD showed a direct correlation between increased adiponectin plasma concentration and cardiovascular mortality [53]. In multivariable adjusted Cox models, 1 µ/mL increase in adiponectin was associated with a 3% (hazard ratio 1.03; 95% CI 1.01 to 1.05; *p* = 0.02) increased risk of all-cause and 6% (hazard ratio 1.06; 95% CI 1.03 to 1.09; *p* < 0.001) increased risk of cardiovascular mortality.

A potential explanation for these apparently conflicting data is the reported association between increased adiponectin concentrations and poor nutritional status in CKD. However, the existence of a link between higher adiponectin levels and increased cardiovascular risk in CKD remains to be clarified. In view of the bidirectional association of adiponectin with events, its role as a useful marker of cardiovascular risk in CKD remains uncertain, pending accumulation of further evidence [16].

## 7. Leptin in Renal Disease

Leptin is a single-chain 16 kDa protein encoded by the obese (*ob*) gene and mainly secreted by adipocytes, although it can also be produced by vascular smooth muscle cells and cardiomyocytes [54]. Its fundamental function is control of the appetite stimulus, regulation of food intake and energy expenditure [55]. Leptin is also a proinflammatory adipocytokine and is primarily cleared by a combination of glomerular filtration and tubular degradation [56,57]. Its levels increase in parallel with insulin levels, glucocorticoids, other cytokines and in obesity [57,58]. In healthy subjects and patients with diabetes mellitus, weight loss has been shown to be accompanied by a decrease in leptin serum concentration [58,59]. These data suggest that high leptin concentrations are a component of metabolic syndrome and may have a role in increasing the cardiovascular risk in these patients. In patients with CKD, leptin levels are elevated, particularly in those with ESKD on dialysis [60,61].

A number of factors affect the metabolism of leptin beyond reduced renal clearance. Among others, metabolic acidosis and uremic factors not better identified reduce the gene expression for leptin [62].

Leptin has been associated with markers of vascular disease such as decreased arterial distensibility and increased carotid intima-media thickness [63,64]. In a small cohort of kidney transplant recipients (*n* = 74), there was a positive association between elevated serum leptin levels and increased peripheral arterial stiffness [65]. Aguilera et al. described an association between leptin levels and left ventricular hypertrophy in a small peritoneal dialysis cohort [66]. More recently, Noor et al. showed that leptin and C-reactive protein levels increased significantly with progression of CKD [67]. Kastarinen et al. reported that mean serum leptin levels were associated with atherogenic lipid profiles [68].

Currently the data on the role of leptin as a promoter of atherosclerosis in CKD are limited and at times conflicting. Scholze et al. reported an association of leptin serum levels with cardiovascular events in 71 prevalent hemodialysis patients followed for 83 months [69]. Event-free survivors had higher levels of baseline leptin than that of patients who suffered a lethal cardiovascular event (7.7 ± 7.8 microg/L vs 4.7 ± 9.4 microg/L; *p* = 0.003). In addition, patients with a leptin serum level below the median (< 2.6 microg/L) had a shorter life expectancy than those with a serum level above the median. In two other studies, leptin was not a significant predictor of all-cause mortality and cardiovascular morbidity in hemodialysis patients [70,71]. Hence, the value of leptin as a marker of risk remains unclear in CKD.

## 8. Therapeutic Approaches

In patients from the general population, regression of EAT volume has been attained with changes in lifestyle and medical interventions. Nakazato et al. [72] showed a 2% regression in EAT volume in 54 patients who lost 5% of their initial body weight in 4 y, compared to a 23% increase in 71 subjects who gained weight. Two groups of investigations reported that EAT volume decreased after intensive lipid-lowering therapy with statins [73,74]. The latter may have occurred because of the known anti-inflammatory activity and inhibition of vasa-vasorum proliferation by statins, which are believed to directly contribute to the development of atherosclerosis [75]. In patients with diabetes mellitus, the addition of an inhibitor of dipeptidyl peptidase-4 (DPP-4) to other baseline therapies produced a significant reduction in EAT volume during a 24 w follow-up [76]. Similar benefits were obtained with pioglitazone in patients with the metabolic syndrome [77]. In patients with CKD, the noncalcium-based phosphate binding agent sevelamer has been shown to reduce serum cholesterol and markers of inflammation [78]. Additionally, sevelamer slowed the progression of CAC and EAT in hemodialysis patients [78]. Whether weight loss and regular aerobic exercise may reduce the accumulation of visceral adipose tissue in patients with CKD remains to be addressed in prospective studies.

## 9. Conclusions

The extreme cardiovascular risk of patients with CKD is not entirely explained by traditional risk factors. While calcification of the cardiovascular system may be an important contributor to this increased risk, other cardiometabolic factors very likely add to the burden of disease. A strong association between EAT and cardiovascular disease has been established in patients with normal renal function. A similar link in patients with CKD is very likely, and although not yet fully proven, it is quickly unraveling. Adiponectin and leptin with their paracrine and endocrine effects are probably important actors in this scenario. Ongoing research may clarify whether EAT and the adipocytokines secreted by other visceral adipose tissue are active participants in the development of the frequent complications of CKD. Animal models could potentially be of help to elucidate some of the links between adipocytokines and cardiovascular disease. Unfortunately, experimental animals have little to no EAT and visceral tissue and the knowledge collected so far is limited (Table 2).

## Figures and Tables

**Table 1:** Proposed pathophysiological effects of adipocytokines produced in human visceral adipose tissue [25].

Adipokine	Metabolism in CKD		Cardiovascular Effects				
	Accumulation	Outcome	Oxidative Stress	Ischemia/Reperfusion	LV Hypertrophy	Remodeling	Inflammation
Adiponectin	Yes	Inflammation/CVD	⇩	⇩	⇩	⇩	⇩
Leptin	Yes	Inflammation/CVD	⇧	⇩	⇧	⇧	⇧
Visfatin	Yes	endothelial damage/lipid dysregulation/CVD	⇧	⇧	⇩	⇩	⇧
Apelin	Yes	Inflammation/CVD	⇩	⇩	U	U	⇩
Resistin	Yes	endothelial damage/inflammation/CVD	⇧	⇧	⇧	⇧	⇧
Omentin	Yes	endothelial damage/inflammation/CVD	⇩	⇩	U	U	⇩

Arrows down: decreased. Arrows up: increased. CKD, chronic kidney disease; CVD, cardiovascular disease; LV, left ventricle; U, unknown.

**Table 2:** Animal experiments supporting the role of epicardial adipose tissue in atherosclerosis and inflammation.

Publication	Experimental Animal	Outcome
Marchington et al. [79]	Guinea pigs	Epicardial adipose tissue stores energy and protects the coronary circulation from elevated fatty acid levels
Ishikawa et al. [80]	Rabbits	In cholesterol-fed animals, atherosclerosis does not develop in coronary artery segments embedded in myocardial bridges, but only in segments surrounded by epicardial adipose tissue
Wang et al. [81]	New Zealand white rabbits	A high-fat diet induces a phenotype conversion in the epicardial adipose tissue from brown to white adipose tissue with focal development of atherosclerosis and progressive increase of leptin mRNA and IL-6 expression.
Bale et al. [82]	Mice	Mouse pericardial fat has the characteristics of visceral fat and is regulated by pregnancy-associated plasma protein-A (PAPP-A) that affects insulin sensitivity.
Wu et al. [83]	Mice	The pericardial adipose tissue has a higher concentration of IL-10-producing B cells than other adipose tissues, and these cells have anti-inflammatory activity following myocardial infarction.

## References

[1] Russo R., Di Iorio B., Di Lullo L., Russo D. (2018). Epicardial adipose tissue: New parameter for cardiovascular risk assessment in high risk populations. J. Nephrol..

[2] Bornachea O., Vea A., Llorente-Cortes V. (2018). Interplay between epicardial adipose tissue, metabolic and cardiovascular diseases. Clin. Investig. Arterioscler..

[3] Singh N., Singh H., Khanijoun H.K., Iacobellis G. (2007). Echocardiographic assessment of epicardial adipose tissue—A marker of visceral adiposity. Mcgill. J. Med..

[4] Janik M., Hartlage G., Alexopoulos N., Mirzoyev Z., McLean D.S., Arepalli C.D., Chen Z., Stillman A.E., Raggi P. (2010). Epicardial adipose tissue volume and coronary artery calcium to predict myocardial ischemia on positron emission tomography-computed tomography studies. J. Nucl. Cardiol..

[5] Nelson A.J., Worthley M.I., Psaltis P.J., Carbone A., Dundon B.K., Duncan R.F., Piantadosi C., Lau D.H., Sanders P., Wittert G.A. (2009). Validation of cardiovascular magnetic resonance assessment of pericardial adipose tissue volume. J. Cardiovasc. Magn. Reson..

[6] Alexopoulos N., McLean D.S., Janik M., Arepalli C.D., Stillman A.E., Raggi P. (2010). Epicardial adipose tissue and coronary artery plaque characteristics. Atherosclerosis.

[7] Nerlekar N., Brown A.J., Muthalaly R.G., Talman A., Hettige T., Cameron J.D., Wong D.T.L. (2017). Association of epicardial adipose tissue and high-risk plaque characteristics: A systematic review and meta-analysis. J. Am. Heart Assoc..

[8] Bachar G.N., Dicker D., Kornowski R., Atar E. (2012). Epicardial adipose tissue as a predictor of coronary artery disease in asymptomatic subjects. Am. J. Cardiol..

[9] Ding J., Hsu F.-C., Harris T.B., Liu Y., Kritchevsky S.B., Szklo M., Ouyang P., Espeland M.A., Lohman K.K., Criqui M.H. (2009). The association of pericardial fat with incident coronary heart disease: The Multi-Ethnic Study of Atherosclerosis (MESA). Am. J. Clin. Nutr..

[10] Turkmen K., Kayikcioglu H., Ozbek O., Solak Y., Kayrak M., Samur C., Anil M., Zeki Tonbul H. (2011). The relationship between epicardial adipose tissue and malnutrition, inflammation, atherosclerosis/calcification syndrome in ESRD patients. Clin. J. Am. Soc. Nephrol..

[11] Cordeiro A.C., Amparo F.C., Oliveira M.A.C., Amodeo C., Smanio P., Pinto I.M., Lindholm B., Stenvinkel P., Carrero J.J. (2015). Epicardial fat accumulation, cardiometabolic profile and cardiovascular events in patients with stages 3-5 chronic kidney disease. J. Intern. Med..

[12] Gansevoort R.T., Correa-Rotter R., Hemmelgarn B.R., Jafar T.H., Heerspink H.J.L., Mann J.F., Matsushita K., Wen C.P. (2013). Chronic kidney disease and cardiovascular risk: Epidemiology, mechanisms, and prevention. Lancet.

[13] Sarnak M.J., Levey A.S., Schoolwerth A.C., Coresh J., Culleton B., Hamm L.L., McCullough P.A., Kasiske B.L., Kelepouris E., Klag M.J. (2003). Kidney disease as a risk factor for development of cardiovascular disease: A statement from the American Heart Association councils on kidney in cardiovascular disease, high blood pressure research, clinical cardiology, and epidemiology and prevention. Hypertension.

[14] Levin A., Rigatto C., Brendan B., Madore F., Muirhead N., Holmes D. (2013). Cohort profile: Canadian study of prediction of death, dialysis and interim cardiovascular events (CanPREDDICT ). BMC Nephrol..

[15] Berl T., Henrich W. (2006). Kidney-heart interactions: Epidemiology, pathogenesis, and treatment. Clin. J. Am. Soc. Nephrol..

[16] D’Marco L., Bellasi A., Raggi P. (2015). Cardiovascular biomarkers in chronic kidney disease: State of current research and clinical applicability. Dis. Markers.

[17] Vickery S., Webb M.C., Price C.P., John R.I., Abbas N.A., Lamb E.J. (2008). Prognostic value of cardiac biomarkers for death in a non-dialysis chronic kidney disease population. Nephrol. Dial. Transplant..

[18] Rabkin S.W. (2007). Epicardial fat: Properties, function and relationship to obesity. Obes. Rev..

[19] Iacobellis G., Bianco A.C. (2011). Epicardial adipose tissue: Emerging physiological, pathophysiological and clinical features. Trends Endocrinol. Metab..

[20] Marchington J.M., Mattacks C.A., Pond C.M. (1989). Adipose tissue in the mammalian heart and pericardium: Structure, foetal development and biochemical properties. Comp. Biochem. Physiol. B..

[21] Corradi D., Maestri R., Callegari S., Pastori P., Goldoni M., Luong T.V., Bordi C. (2004). The ventricular epicardial fat is related to the myocardial mass in normal, ischemic and hypertrophic hearts. Cardiovasc. Pathol..

[22] Pezeshkian M., Noori M., Najjarpour-Jabbari H., Abolfathi A., Darabi M., Darabi M., Darabi M., Shaaker M., Shahmohammadi G. (2009). Fatty acid composition of epicardial and subcutaneous human adipose tissue. Metab. Syndr. Relat. Disord..

[23] Sacks H.S., Fain J.N., Holman B., Cheema P., Chary A., Parks F., Karas J., Optican R., Bahouth S.W., Garrett E. (2009). Uncoupling protein-1 and related messenger ribonucleic acids in human epicardial and other adipose tissues: Epicardial fat functioning as brown fat. J. Clin. Endocrinol. Metab..

[24] Iacobellis G. (2009). Epicardial and pericardial fat: Close, but very different. Obesity.

[25] Akoumianakis I., Antoniades C. (2017). The interplay between adipose tissue and the cardiovascular system: Is fat always bad?. Cardiovasc. Res..

[26] Turer A.T., Scherer P.E. (2012). Adiponectin: Mechanistic insights and clinical implications. Diabetologia.

[27] Salazar J., Luzardo E., Mejías J.C., Rojas J., Ferreira A., Rivas-Ríos J.R., Bermúdez V. (2016). Epicardial Fat: Physiological, Pathological, and Therapeutic Implications. Cardiol. Res. Pract..

[28] Ouchi N., Parker J.L., Lugus J.J., Walsh K. (2011). Adipokines in inflammation and metabolic disease. Nat. Rev. Immunol..

[29] Kaisar O.M., Johnson D.W., Prins J.B., Isbel N. (2008). The role of novel biomarkers of cardiovascular disease in chronic kidney disease: Focus on adiponectin and leptin. Curr. Cardiol. Rev..

[30] Wong H.K., Cheung T.T., Cheung B.M.Y. (2012). Adrenomedullin and cardiovascular diseases. JRSM Cardiovasc. Dis..

[31] Scholze A., Tepel M. (2007). Role of leptin in reverse epidemiology in chronic kidney disease. Semin. Dial..

[32] Ueno K., Anzai T., Jinzaki M., Yamada M., Jo Y., Maekawa Y., Kawamura A., Yoshikawa T., Tanami Y., Sato K. (2009). Increased Epicardial Fat Volume Quantified by 64-Multidetector Computed Tomography is Associated With Coronary Atherosclerosis and Totally Occlusive Lesions. Circ. J..

[33] Reinhardt M., Cushman T.R., Thearle M.S., Krakoff J. (2019). Epicardial adipose tissue is a predictor of decreased kidney function and coronary artery calcification in youth- and early adult onset type 2 diabetes mellitus. J. Endocrinol. Investig..

[34] Nakanishi K., Fukuda S., Tanaka A., Otsuka K., Taguchi H., Yoshikawa J., Shimada K. (2015). Epicardial adipose tissue accumulation is associated with renal dysfunction and coronary plaque morphology on multidetector computed tomography. Circ. J..

[35] Karohl C., D’Marco L., Bellasi A., Raggi P. (2013). Hybrid myocardial imaging for risk stratification prior to kidney transplantation: Added value of coronary calcium and epicardial adipose tissue. J. Nucl. Cardiol..

[36] D’Marco L.G., Bellasi A., Kim S., Chen Z., Block G.A., Raggi P. (2013). Epicardial adipose tissue predicts mortality in incident hemodialysis patients: A substudy of the Renagel in New Dialysis trial. Nephrol. Dial. Transplant..

[37] Iacobellis G., Pistilli D., Gucciardo M., Leonetti F., Miraldi F., Brancaccio G., Gallo P., di Gioia C.R. (2005). Adiponectin expression in human epicardial adipose tissue in vivo is lower in patients with coronary artery disease. Cytokine.

[38] Cheng K.H., Chu C.S., Lee K.T., Lin T.H., Hsieh C.C., Chiu C.C., Voon W.C., Sheu S.H., Lai W.T. (2008). Adipocytokines and proinflammatory mediators from abdominal and epicardial adipose tissue in patients with coronary artery disease. Int. J. Obes..

[39] Mazurek T., Zhang L.F., Zalewski A., Mannion J.D., Diehl J.T., Arafat H., Sarov-Blat L., O’Brien S., Keiper E.A., Johnson A.G. (2003). Human epicardial adipose tissue is a source of inflammatory mediators. Circulation.

[40] Ouchi N., Kihara S., Arita Y., Okamoto Y., Maeda K., Kuriyama H., Hotta K., Nishida M., Takahashi M., Muraguchi M. (2000). Adiponectin, an adipocyte-derived plasma protein, inhibits endothelial NF-kappaB signaling through a cAMP-dependent pathway. Circulation.

[41] Baker A.R., Silva N.F., da Quinn D.W., Harte A.L., Pagano D., Bonser R.S., Kumar S., McTernan P.G. (2006). Human epicardial adipose tissue expresses a pathogenic profile of adipocytokines in patients with cardiovascular disease. Cardiovasc. Diabetol..

[42] Ghantous C.M., Azrak Z., Hanache S., Abou-Kheir W., Zeidan A. (2015). Differential role of leptin and adiponectin in cardiovascular system. Int. J. Endocrinol..

[43] Kershaw E.E., Flier J.S. (2004). Adipose tissue as an endocrine organ. J. Clin. Endocrinol. Metab..

[44] Trujillo M.E., Sullivan S., Harten I., Schneider S.H., Greenberg A.S., Fried S.K. (2004). Interleukin-6 regulates human adipose tissue lipid metabolism and leptin production in vitro. J. Clin. Endocrinol. Metab..

[45] Fisher F.F.M., Trujillo M.E., Hanif W., Barnett A.H., McTernan P.G., Scherer P.E., Kumar S. (2005). Serum high molecular weight complex of adiponectin correlates better with glucose tolerance than total serum adiponectin in Indo-Asian males. Diabetologia.

[46] Bouskila M., Pajvani U.B., Scherer P.E. (2005). Adiponectin: A relevant player in PPARgamma-agonist-mediated improvements in hepatic insulin sensitivity?. Int. J. Obes..

[47] Whitehead J.P., Richards A.A., Hickman I.J., Macdonald G.A., Prins J.B. (2006). Adiponectin—A key adipokine in the metabolic syndrome. Diabetes Obes. Metab..

[48] Komura N., Kihara S., Sonoda M., Maeda N., Tochino Y., Funahashi T., Shimomura I. (2010). Increment and impairment of adiponectin in renal failure. Cardiovasc. Res..

[49] Kumada M., Kihara S., Sumitsuji S., Kawamoto T., Matsumoto S., Ouchi N., Arita Y., Okamoto Y., Shimomura I., Hiraoka H. (2003). Association of hypoadiponectinemia with coronary artery disease in men. Arterioscler. Thromb. Vasc. Biol..

[50] Zoccali C., Mallamaci F. (2011). Adiponectin and leptin in chronic kidney disease: Causal factors or mere risk markers?. J. Ren. Nutr..

[51] Zoccali C., Mallamaci F., Tripepi G., Benedetto F.A., Cutrupi S., Parlongo S., Malatino L.S., Bonanno G., Seminara G., Rapisarda F. (2002). Adiponectin, metabolic risk factors, and cardiovascular events among patients with end-stage renal disease. J. Am. Soc. Nephrol..

[52] Becker B., Kronenberg F., Kielstein J.T., Haller H., Morath C. (2005). Renal insulin resistance syndrome, adiponectin and cardiovascular events in patients with kidney disease: The Mild and Moderate Kidney Disease Study. J. Am. Soc. Nephrol..

[53] Menon V., Li L., Wang X., Greene T., Balakrishnan V., Madero M., Pereira A.A., Beck G.J., Kusek J.W., Collins A.J. (2006). Adiponectin and mortality in patients with chronic kidney disease. J. Am. Soc. Nephrol..

[54] Nisoli E., Tonello C., Briscini L., Flaim R., Carruba M.O. (1996). Leptin and nerve growth factor regulate adipose tissue. Nat. Med..

[55] Stenvinkel P. (1998). Leptin—A new hormone of definite interest for the nephrologist. Nephrol. Dial. Transplant..

[56] Fried S.K., Ricci M.R., Russell C.D., Laferrère B. (2000). Regulation of leptin production in humans. J. Nutr..

[57] Considine R.V., Sinha M.K., Heiman M.L., Kriauciunas A., Stephens T.W., Nyce M.R., Ohannesian J.P., Marco C.C., McKee L.J., Bauer T.L. (1996). Serum immunoreactive-leptin concentrations in normal-weight and obese humans. N. Engl. J. Med..

[58] Maffei M., Halaas J., Ravussin E., Pratley R.E., Lee G.H., Zhang Y., Fei H., Kim S., Lallone R., Ranganathan S. (1995). Leptin levels in human and rodent: Measurement of plasma leptin and ob RNA in obese and weight-reduced subjects. Nat. Med..

[59] Boden G., Sargrad K., Homko C., Mozzoli M., Stein T.P. (2005). Effect of a low-carbohydrate diet on appetite, blood glucose levels, and insulin resistance in obese patients with type 2 diabetes. Ann. Intern. Med..

[60] Merabet E., Dagogo-Jack S., Coyne D.W., Klein S., Santiago J.V., Hmiel S.P., Landt M. (1997). Increased plasma leptin concentration in end-stage renal disease. J. Clin. Endocrinol. Metab..

[61] Díez J.J., Iglesias P., Fernández-Reyes M.J., Aguilera A., Bajo M.A., Alvarez-Fidalgo P., Codoceo R., Selgas R. (2005). Serum concentrations of leptin, adiponectin and resistin, and their relationship with cardiovascular disease in patients with end-stage renal disease. Clin. Endocrinol..

[62] Teta D., Bevington A., Brown J., Pawluczyk I., Harris K., Walls J. (2003). Acidosis downregulates leptin production from cultured adipocytes through a glucose transport-dependent post-transcriptional mechanism. J. Am. Soc. Nephrol..

[63] Ciccone M., Vettor R., Pannacciulli N., Minenna A., Bellacicco M., Rizzon P., Giorgino R., De Pergola G. (2001). Plasma leptin is independently associated with the intima-media thickness of the common carotid artery. Int. J. Obes. Relat. Metab. Disord..

[64] Singhal A., Farooqi I.S., Cole T.J., Rahilly S.O., Fewtrell M., Kattenhorn M., Lucas A., Deanfield J. (2002). Influence of leptin on arterial distensibility. Circulation.

[65] Lee M.-C., Chen Y.-C., Ho G.-J., Shih M.-H., Chou K.-C., Hsu B.-G. (2014). Serum leptin levels positively correlate with peripheral arterial stiffness in kidney transplantation patients. Transplant. Proc..

[66] Aguilera A., Bajo M.A., Rebollo F., Díez J.J., Díaz C., Paiva A., Codoceo R., Selgas R. (2002). Leptin as a marker of nutrition and cardiovascular risk in peritoneal dialysis patients. Adv. Perit. Dial..

[67] Noor S., Alam F., Fatima S.S., Khan M., Rehman R. (2018). Role of Leptin and dyslipidemia in chronic kidney disease. Pak. J. Pharm. Sci..

[68] Kastarinen H., Kesäniemi Y.A., Ukkola O. (2009). Leptin and lipid metabolism in chronic kidney failure. Scand. J. Clin. Lab. Investig..

[69] Scholze A., Rattensperger D., Zidek W., Tepel M. (2007). Low serum leptin predicts mortality in patients with chronic kidney disease stage 5. Obesity.

[70] Beberashvili I., Sinuani I., Azar A., Yasur H., Feldman L., Averbukh Z., Weissgarten J. (2011). Longitudinal study of leptin levels in chronic hemodialysis patients. Nutr. J..

[71] Tsai Y.-C., Lee C.-T., Huang T.-L., Cheng B.-C., Kuo C.-C., Su Y., Ng H.Y., Yang C.C., Chuang F.R., Liao S.C. (2007). Inflammatory marker but not adipokine predicts mortality among long-term hemodialysis patients. Mediators. Inflamm..

[72] Nakazato R., Rajani R., Cheng V.Y., Shmilovich H., Nakanishi R., Otaki Y., Gransar H., Slomka P.J., Hayes S.W., Thomson L.E. (2012). Weight change modulates epicardial fat burden: A 4-year serial study with non-contrast computed tomography. Atherosclerosis.

[73] Parisi V., Petraglia L., D’Esposito V., Cabaro S., Rengo G., Caruso A., Grimaldi M.G., Baldascino F., De Bellis A., Vitale D. (2019). Statin therapy modulates thickness and inflammatory profile of human epicardial adipose tissue. Int. J. Cardiol..

[74] Alexopoulos N., Melek B.H., Arepalli C.D., Hartlage G.-R., Chen Z., Kim S., Stillman A.E., Raggi P. (2013). Effect of intensive versus moderate lipid-lowering therapy on epicardial adipose tissue in hyperlipidemic post-menopausal women: A substudy of the BELLES trial (Beyond Endorsed Lipid Lowering with EBT Scanning). J. Am. Coll. Cardiol..

[75] Subbotin V.M. (2012). Neovascularization of coronary tunica intima (DIT) is the cause of coronary atherosclerosis. Lipoproteins invade coronary intima via neovascularization from adventitial vasa vasorum, but not from the arterial lumen: A hypothesis. Theor. Biol. Med. Model..

[76] Lima-Martínez M.M., Paoli M., Rodney M., Balladares N., Contreras M., D’Marco L., Iacobellis G. (2016). Effect of sitagliptin on epicardial fat thickness in subjects with type 2 diabetes and obesity: A pilot study. Endocrine.

[77] Sacks H.S., Fain J.N., Cheema P., Bahouth S.W., Garrett E., Wolf R.Y., Wolford D., Samaha J. (2011). Inflammatory genes in epicardial fat contiguous with coronary atherosclerosis in the metabolic syndrome and type 2 diabetes: Changes associated with pioglitazone. Diabetes Care.

[78] Ko S.M., Zhang C., Chen Z., D’Marco L., Bellasi A., Stillman A.E., Block G., Raggi P. (2016). Epicardial adipose tissue volume increase in hemodialysis patients treated with sevelamer or calcium-based phosphate binders: A substudy of the Renagel in new dialysis trial. J. Nephrol..

[79] Marchington J.M., Pond C.M. (1990). Site-specific properties of pericardial and epicardial adipose tissue: The effects of insulin and high-fat feeding on lipogenesis and the incorporation of fatty acids in vitro. Int. J. Obes..

[80] Ishikawa Y., Ishii T., Asuwa N., Masuda S. (1997). Absence of atherosclerosis evolution in the coronary arterial segment covered by myocardial tissue in cholesterol-fed rabbits. Virchows Arch..

[81] Wang J., Chen D., Cheng X.M., Zhang Q.G., Peng Y.P., Wang L.J., He S.Q., Gong J.B. (2015). Influence of phenotype conversion of epicardial adipocytes on the coronary atherosclerosis and its potential molecular mechanism. Am. J. Transl. Res..

[82] Bale L.K., West S.A., Conover C.A. (2018). Characterization of mouse pericardial fat: Regulation by PAPP-A. Growth. Horm. IGF Res..

[83] Wu L., Dalal R., Cao C.D., Postoak J.L., Yang G., Zhang Q., Wang Z., Lal H., Van Kaer L. (2019). IL-10-producing B cells are enriched in murine pericardial adipose tissues and ameliorate the outcome of acute myocardial infarction. Proc. Natl. Acad. Sci. USA.

